# Unlikely heroes on the long and winding road to potato inbreeding

**DOI:** 10.1007/s42994-023-00109-5

**Published:** 2023-07-18

**Authors:** Luca Comai

**Affiliations:** Department of Plant Biology and Genome Center, University of California Davis, Davis, CA 95616 USA

**Keywords:** Hybrid seed, Deleterious alleles, Inbreeding, Genetic load, Clonal propagation

## Abstract

Conversion of potato from a tetraploid, heterozygous, vegetatively propagated crop to a diploid F1 hybrid, propagated via botanical seed, would constitute a considerable advance for global agriculture, but faces multiple challenges. One such challenge is the difficulty in inbreeding potato, which involves purging deleterious alleles from its genome. This commentary discusses possible reasons for this difficulty and highlights a recent sequence-based effort to classify SNP variation, in potato germplasm, according to its deleterious potential. Tools and strategies connected to this database may facilitate development of F1 hybrids.

About one hundred and fifty years ago, Darwin studied inbreeding in Ipomea, a species related to potato that displays strong inbreeding depression. After considerable effort he identified a rare vigorous plant, in the sixth inbreeding generation, which he called Hero (Darwin 1876). Facing a similar inbreeding challenge, ongoing efforts aim to convert potato from a tetraploid, heterozygous, vegetatively propagated crop to a diploid F1 hybrid, propagated via botanical seed. This is a deserving effort, but it is proving to be quite difficult. But first some background. Genetically speaking there are two types of crops: homozygous or heterozygous. Take, for example, soybean and (traditional) rice: they are selfers, with high homozygosity, and are propagated through botanical seeds. The major advantage of homozygosity is that progeny from self-pollination resemble the parent and each other, providing farmers with uniform and predictable crops. Many economical species, including potato, on the other hand, are heterozygous. This state can increase yield and stress tolerance, an advantage known as heterosis (Birchler et al. 2010; Lippman and Zamir 2007). At the same time, heterozygosity has a significant drawback: the progeny of heterozygous parents are variable and, therefore, not suitable for large-scale agriculture. The meioses necessary to produce true botanical seeds scramble the ideal combination of alleles that make an adapted variety.

A practical solution entails vegetative propagation (cloning), which is employed in most tree crops, grapes, mints, cassava, sweet potato, and potato. Propagation by cuttings, or tubers, ‘freezes’ the genome of the rare individuals that display unique and valuable properties. A clone, surprisingly, is not a complete dead end: clonal evolution, or sporting, can be an effective way to improve a variety (Foster and Aranzana 2018): for example, the various sweet orange varieties are clonal derivatives of a single, ancient individual (Wang et al. 2021). Cloning, however, has disadvantages. The annual scaling up of seed potatoes is an expensive process, as the tubers are bulky and perishable, and therefore burdensome to collect, ship, and store.

Vegetative propagation also results in progressive accumulation of yield-depressing pathogens. In tree species, these problems are offset by the plants’ long life. Cloning, however, is cumbersome for annual crops such as potato. The current method for breeding potatoes is well established (Jansky and Spooner 2018): the breeder selects and crosses interesting parents, screens many progeny to eventually select a rare, optimal plant that becomes a new variety to be grown, via clonal propagation. How many progeny should one screen? It helps to be lucky: Luther Burbank famously selected variety, Burbank, from the few seeds found in a spontaneous single berry formed on variety Early Rose (Bethke et al. 2014). A few years later, an unknown farmer identified a valuable Burbank sport, now known as Russet Burbank, a major cultivar still predominant in the US over a hundred years later (Bethke et al. 2014).

Heterozygosity can be exploited: If inbred lines and an efficient hybridization method are available, F1 hybrids can provide significant advantages (Birchler et al. 2010; Crow 1998). Hybrid breeding is responsible for the great yield progress made with maize and is considered the pinnacle of breeding technology (Duvick 2005). Hybrid seed is becoming widespread in rice, and it is a desirable target in multiple other crops (Huang et al. 2016). Everything else being the same, implementation of a true seed system in potato would benefit both industrialized and small-scale farmers (Jansky et al. 2016).

Why has this conversion not happened in over a hundred years of modern potato breeding? Two main reasons. First, inbreeding is difficult (Jansky and Spooner 2018). Second, the current potato system has some advantages: it is a successful crop in many countries and its yield/ha, while increasing slowly, can still be optimized by agronomic practices (Haverkort and Struik 2015). Its autotetraploid, heterozygous genome (four equivalent genomic chromosome sets) can harbor considerable variation (Tang et al. 2022; Hardigan et al. 2017) and may increase heterosis (Bingham et al. 1994; Washburn et al. 2019). Importantly, heterozygosity can mask deleterious recessive alleles.

Notwithstanding the above advantages, the arguments for transitioning to a diploid inbred or hybrid system are convincing (Lindhout et al. 2011; Jansky et al. 2016). But what will this entail? Fundamentally, drastic inbreeding and selection of optimal hybrid parents. But, here, the devil is in the details. Three problems arise: first, the source of diploids; second, self-incompatibility; and third, inbreeding depression, presumably through the uncovering of recessive deleterious alleles. Considerable progress has been made addressing the first two problems. First, there is high diversity of potato landraces, many diploid, in the Andean region from which promising diploids can be recruited into breeding programs (Lindhout et al. 2011; Jansky et al. 2016). Furthermore, diploids can be extracted by crossing a 4X potato to a specialized 2X haploid inducer (Hougas and Peloquin 1958; Hermsen and Verdenius 1973). Second, the genes responsible for self-incompatibility are known and can be modified, by genome editing or introgression, to achieve self-compatibility (Ye et al. 2018; Lindhout et al. 2011).

The last requirement, inbreeding to homozygosity, poses a significant challenge. In other heterozygous cultivated species, such as maize and beet, inbreeding was successful resulting in fixed inbred lines that yield high heterosis when the right parents are hybridized (Crow 1998). The method entails selfing, growing many progeny, selecting, and repeating as many times as necessary (Willis 1999; Roessler et al. 2019). Yet, while potato inbreeding has been attempted for decades, the results are underwhelming. Sequencing Solyntus, an F9 predicted to have less than 1% heterozygosity, revealed instead that 20% of its genome is heterozygous (van Lieshout et al. 2020). Similar, unexpected levels of heterozygosity were documented in other pedigrees (Marand et al. 2019).

Why then are adapted potato inbreds so difficult to achieve? A plausible explanation is that the potato genome harbors many deleterious recessive alleles (Lindhout et al. 2011; Crnokrak and Barrett 2002). Upon selfing, these alleles become uncovered and must be purged through selection. For this to be probable, however, two conditions must be met. First, each deleterious allele must be amenable to artificial selection; i.e., it must have a detectable effect on phenotype. Second, recombination must be such that adapted haplotypes can emerge on most chromosomes. Purging a few, highly deleterious alleles is relatively simple (Lande and Schemske 1985; Charlesworth and Charlesworth 1999). Zhang et al. (2021) developed a genome design pipeline for identifying and eliminating large-effect deleterious alleles, but the task becomes difficult when there are many slightly deleterious alleles, which are often linked in repulsion (Fig. 1), a phenomenon identified over half a century ago and called the Hill–Robertson effect (Comeron et al. 2008; Hill and Robertson 1966). This effect may explain the higher-than-expected retention of heterozygosity in inbreeding experiments (Roessler et al. 2019; van Lieshout et al. 2020; Wu et al. 2023; Marand et al. 2019).

**Fig. 1:**
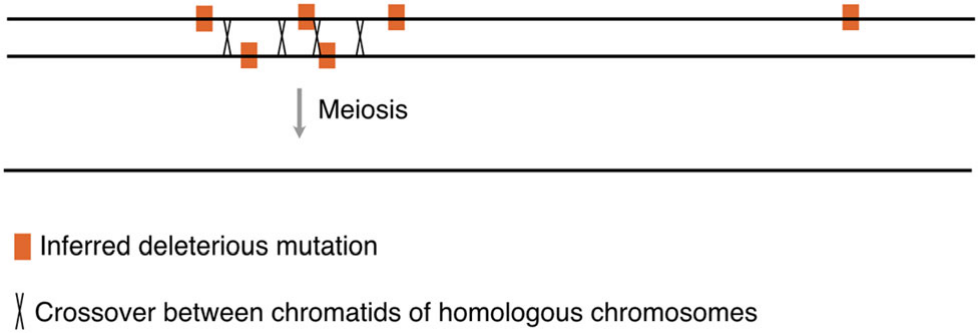
Purging of deleterious alleles. The image illustrates the recombination required to produce meiotic products free of deleterious alleles. The difficulty in breaking repulsion linkage arrangements has been described as the Hill-Robertson effect

Knowledge of deleterious mutations coupled to genomic selection should facilitate the breeding of suitable inbreds. In a recent paper, Wu et al. (2023) scored the deleterious allele burden carried by potato germplasm and segregating families. They used a method called Genomic Evolutionary Rate Profiling (GERP), which at its core, uses a well-established approach: alignment of similar sequences identifies highly conserved positions (Fig. 2). Because purifying selection removes mutations at these sites, changes should be deleterious. First developed in humans (Davydov et al. 2010), the growing adoption of GERP score analysis in plant genomics stems from the power of its computational approach and phylogenetic inference.

**Fig. 2:**
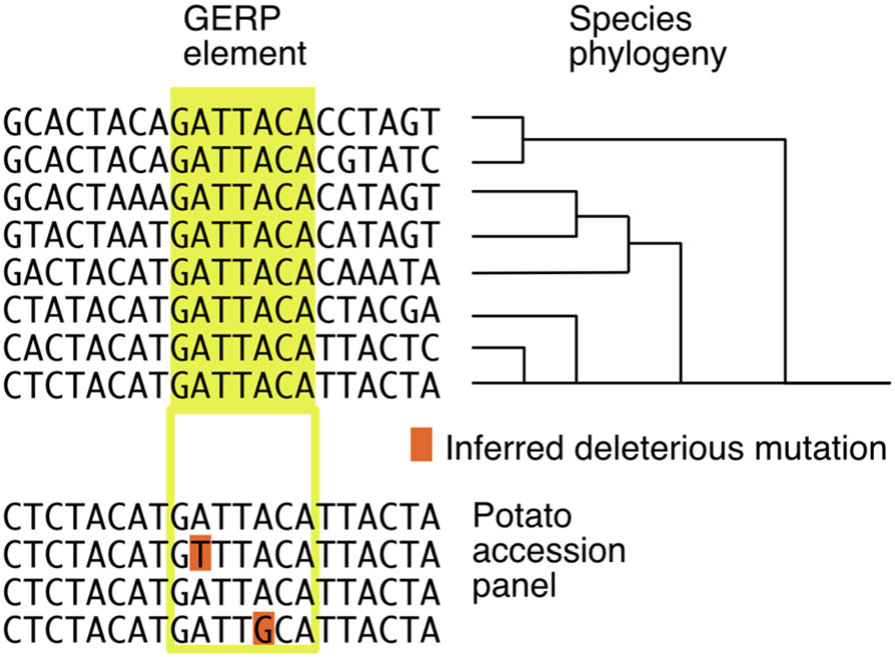
Genomic Evolutionary Rate Profiling. The method identifies homologous regions in the genomes of related species, aligns them, and determines which elements remain invariant, presumably because of purifying selection. Rare population variants occurring in conserved elements are deemed deleterious and assigned a quantitative GERP score, based on inferred significance and severity of the base change

For potato, starting with the genome sequence of 100 Solanales species, Wu et al. (2023) identified sites displaying high to moderate conservation. Most of these sites are in the translated part of the genome (CDS), but a substantial fraction is in introns, in transcribed but untranslated regions (UTRs), in promoters, and even within intergenic DNA. Next, these authors surveyed a panel of 192 potatoes for SNP overlapping conserved sites. Each accession carried hundreds to thousands of these variants, which can be inferred to be relatively recent mutations. Taken individually, each SNP was often rare and, interestingly, about half were in non-coding DNA.

Relating GERP score to plant vigor and genotype, the authors identified a dangerous flaw of visual selection of promising landraces. As inbreeding depression increases proportional to homozygosity, plants that maintain heterozygosity stand out by their desirable characteristics, such as vigor and productivity. Hence, a breeder may, therefore, select these plants assuming they have purged deleterious alleles. However, these apparently promising candidates, can have a higher deleterious allele burden than their weak siblings. This is because their heterozygous regions carry many deleterious variants, in a shielded state. Plants with higher homozygosity may appear less attractive, but often carry fewer overall deleterious alleles. The latter landraces, these authors suggested, should be selected as breeding pedigree parents. This principle appears “counterintuitive" from the point of view of phenotypic selection, but it makes good genetic sense. The same caveat applies during inbreeding when a population traverses a valley of maladaptation. Breeders can be misled by vigor connected to recalcitrant heterozygosity. Not surprisingly, in these heterozygous regions, the authors identified tightly linked deleterious mutations in repulsion linkage. The recombination events necessary to eliminate these deleterious alleles would indeed be very rare.

To evaluate the predictive power of their GERP score analysis, the authors used an F2 population to compare three important vigor-related traits to the deleterious mutation burden. The results were good: yield prediction, for example, improves by about 25%. GERP score SNP burden is not predictive of flowering time, a trait unrelated to vigor. We can conclude that a combination of genotyping and GERP analysis should be useful for genomic-guided inbreeding. Further, the large and systematic classification of conserved sites, and the identification of corresponding rare variants in the potato germplasm, provides a welcome contribution to potato genomics.

Additional information and tools would help the quest for breeding hybrid potatoes. The current model for the deleterious genetic load could be improved. SNPs clearly contribute to the genetic burden, but the current analysis ignores structural variation, which is frequent in plant genomes and known to contribute to phenotype (Alonge et al. 2020; Bastiaanse et al. 2019). In addition, it is not clear what part of the genetic load is recessive. While dominance would be rare, frequent additivity is likely. In other species, deleterious mutations can have an additive effect and even contribute to heterosis (Yang et al. 2017; Willis 1999). A previous study connected potato heterosis to epistatic interactions (Marand et al. 2019). This raises the question of whether alleles with high GERP score could be involved. Last, inbreeding of certain maize genotypes results in loss of transposable elements, suggesting a deleterious role for heterochromatin, which is unlikely to be recessive, but could well have epistatic action (Roessler et al. 2019).

The strategy for inbreeding must be refined. It is likely that potato inbreds will have to carry substantial genetic burden. Breeders will have to compromise between heterotic combining ability (their parental potential to produce superior F1), and their ability to produce sufficient botanical seed for hybrid marketing. A significant variable of the inbreeding equation is the population size needed to achieve a suitable inbred. Multiple generations involving large populations, grown in the field or greenhouse, increase costs proportionally to their size and can become rapidly prohibitive. A solution could be an efficient haploid induction system, which generates inbreds in one or two generations (Jacquier et al. 2020). Currently, potato haploid inducers are used for 4× to 2× conversion and the efficiency varies from 1 to 0.001 per pollination (Amundson et al. 2021; Ordoñez et al. 2021; Busse et al. 2021; Hermsen and Verdenius 1973). If a breeding program could generate thousands of 1 × haploids from 2 × parents, selection for vigor could start with their germination, which requires little space and effort. Lastly, potato breeders should keep an eye on the recent progress in engineering apomixis (Khanday et al. 2019; Conner et al. 2015): clonal seeds, even in 4X varieties, would revolutionize potato breeding (Hermsen 1980).

In conclusion, large scale genomic analysis and the use of genome editing is bringing heavy guns to the inbreeding battle for potato. Careful planning and logistics, and probably new tools will be necessary to fully leverage these approaches toward the Darwinian search for the ultimate potato heroes.

## Data Availability

Data sharing not applicable to this article as no datasets were generated or analysed during the current study.

## References

[CR1] Alonge M (2020). Major impacts of widespread structural variation on gene expression and crop improvement in tomato. Cell.

[CR2] Amundson KR, Ordoñez B, Santayana M, Nganga ML, Henry IM, Bonierbale M, Khan A, Tan EH, Comai L (2021). Rare instances of haploid inducer DNA in potato dihaploids and ploidy-dependent genome instability. Plant Cell.

[CR3] Bastiaanse H, Zinkgraf M, Canning C, Tsai H, Lieberman M, Comai L, Henry I, Groover A (2019). A comprehensive genomic scan reveals gene dosage balance impacts on quantitative traits in Populus trees. Proc Natl Acad Sci.

[CR4] Bethke PC, Nassar AMK, Kubow S, Leclerc YN, Li X-Q, Haroon M, Molen T, Bamberg J, Martin M, Donnelly DJ (2014). History and origin of russet Burbank (netted gem) a sport of Burbank. Am J Potato Res.

[CR5] Bingham ET, Groose RW, Woodfield DR, Kidwell KK (1994). Complementary gene interactions in alfalfa are greater in autotetraploids than diploids. Crop Sci.

[CR6] Birchler JA, Yao H, Chudalayandi S, Vaiman D, Veitia RA (2010). Heterosis. Plant Cell.

[CR7] Busse JS, Jansky SH, Agha HI, Schmitz Carley CA, Shannon LM, Bethke PC (2021). A high throughput method for generating dihaploids from tetraploid potato. Am J Potato Res.

[CR8] Charlesworth B, Charlesworth D (1999). The genetic basis of inbreeding depression. Genet Res.

[CR9] Comeron JM, Williford A, Kliman RM (2008). The Hill-Robertson effect: evolutionary consequences of weak selection and linkage in finite populations. Heredity.

[CR10] Conner JA, Mookkan M, Huo H, Chae K, Ozias-Akins P (2015). A parthenogenesis gene of apomict origin elicits embryo formation from unfertilized eggs in a sexual plant. Proc Natl Acad Sci U S A.

[CR11] Crnokrak P, Barrett SCH (2002). Perspective: purging the genetic load: a review of the experimental evidence. Evolution.

[CR12] Crow JF (1998). 90 years ago: the beginning of hybrid maize. Genetics.

[CR13] Darwin C (1876). The effects of cross and self fertilisation in the vegetable kingdom.

[CR14] Davydov EV, Goode DL, Sirota M, Cooper GM, Sidow A, Batzoglou S (2010). Identifying a high fraction of the human genome to be under selective constraint using GERP++. PLoS Comput Biol.

[CR15] Duvick DN (2005). The contribution of breeding to yield advances in maize (*Zea mays* L.). Advances in agronomy.

[CR16] Foster TM, Aranzana MJ (2018). Attention sports fans! The far-reaching contributions of bud sport mutants to horticulture and plant biology. Hortic Res.

[CR17] Hardigan MA, Laimbeer FPE, Newton L, Crisovan E, Hamilton JP, Vaillancourt B, Wiegert-Rininger K, Wood JC, Douches DS, Farré EM, Veilleux RE, Buell CR (2017). Genome diversity of tuber-bearing Solanum uncovers complex evolutionary history and targets of domestication in the cultivated potato. Proc Natl Acad Sci USA.

[CR18] Haverkort AJ, Struik PC (2015). Yield levels of potato crops: recent achievements and future prospects. Field Crops Res.

[CR19] Hermsen JGT (1980). Breeding for apomixis in potato: Pursuing a utopian scheme. Euphytica.

[CR20] Hermsen J, Verdenius J (1973). Selection from Solanum tuberosum group Phureja of genotypes combining high-frequency haploid induction with homozygosity for embryo-spot. Euphytica.

[CR21] Hill WG, Robertson A (1966). The effect of linkage on limits to artificial selection. Genet Res.

[CR22] Hougas RW, Peloquin SJ (1958). The potential of potato haploids in breeding and genetic research. Am J Potato Res.

[CR23] Huang X (2016). Genomic architecture of heterosis for yield traits in rice. Nature.

[CR24] Jacquier NMA, Gilles LM, Pyott DE, Martinant J-P, Rogowsky PM, Widiez T (2020). Puzzling out plant reproduction by haploid induction for innovations in plant breeding. Nat Plants.

[CR25] Jansky SH, Spooner DM (2018). The evolution of potato breeding. Plant breeding reviews.

[CR26] Jansky SH (2016). Reinventing potato as a diploid inbred line-based crop. Crop Sci.

[CR27] Khanday I, Skinner D, Yang B, Mercier R, Sundaresan V (2019). A male-expressed rice embryogenic trigger redirected for asexual propagation through seeds. Nature.

[CR28] Lande R, Schemske DW (1985). The evolution of self-fertilization and inbreeding depression in plants. I. genetic models. Evolution.

[CR29] Lindhout P, Meijer D, Schotte T, Hutten RCB, Visser RGF, van Eck HJ (2011). Towards F1 hybrid seed potato breeding. Potato Res.

[CR30] Lippman ZB, Zamir D (2007). Heterosis: revisiting the magic. Trends Genet.

[CR31] Marand AP, Jansky SH, Gage JL, Hamernik AJ, de Leon N, Jiang J (2019). Residual heterozygosity and epistatic interactions underlie the complex genetic architecture of yield in diploid potato. Genetics.

[CR32] Ordoñez B, Santayana M, Aponte M, Henry IM, Comai L, Eyzaguirre R, Lindqvist-Kreuze H, Bonierbale M (2021). PL-4 (CIP596131.4): an improved potato haploid inducer. Am J Potato Res.

[CR33] Roessler K, Muyle A, Diez CM, Gaut GRJ, Bousios A, Stitzer MC, Seymour DK, Doebley JF, Liu Q, Gaut BS (2019). The genome-wide dynamics of purging during selfing in maize. Nat Plants.

[CR34] Tang D (2022). Genome evolution and diversity of wild and cultivated potatoes. Nature.

[CR35] van Lieshout N, van der Burgt A, de Vries ME, Ter Maat M, Eickholt D, Esselink D, van Kaauwen MPW, Kodde LP, Visser RGF, Lindhout P, Finkers R (2020). Solyntus, the new highly contiguous reference genome for potato (*Solanum tuberosum*). G3.

[CR36] Wang L (2021). Somatic variations led to the selection of acidic and acidless orange cultivars. Nat Plants.

[CR37] Washburn JD, McElfresh MJ, Birchler JA (2019). Progressive heterosis in genetically defined tetraploid maize. J Genet Genomics.

[CR38] Willis JH (1999). Inbreeding load, average dominance and the mutation rate for mildly deleterious alleles in Mimulus guttatus. Genetics.

[CR39] Wu Y (2023). Phylogenomic discovery of deleterious mutations facilitates hybrid potato breeding. Cell.

[CR40] Yang J, Mezmouk S, Baumgarten A, Buckler ES, Guill KE, McMullen MD, Mumm RH, Ross-Ibarra J (2017). Incomplete dominance of deleterious alleles contributes substantially to trait variation and heterosis in maize. PLoS Genet.

[CR41] Ye M, Peng Z, Tang D, Yang Z, Li D, Xu Y, Zhang C, Huang S (2018). Generation of self-compatible diploid potato by knockout of S-RNase. Nat Plants.

[CR42] Zhang C, Yang Z, Tang D, Zhu Y, Wang P, Li D, Zhu G, Xiong X, Shang Y, Li C, Huang S (2021). Genome design of hybrid potato. Cell.

